# Alterations in the gut microbiome and metabolism profiles reveal the possible molecular mechanism of renal injury induced by hyperuricemia in a mouse model of renal insufficiency

**DOI:** 10.1080/0886022X.2024.2387429

**Published:** 2024-08-12

**Authors:** Ping Liu, Jianli Yang, Meiping Jin, Ping Hu, Yifan Zhu, Yuyan Tang, Yu Chen, Xudong Xu, Haidong He

**Affiliations:** a Division of Nephrology, Minhang Hospital, Fudan University, Shanghai, China; b East China University of Science and Technology, Shanghai, China

**Keywords:** Chronic kidney disease, hyperuricemia, renal injury, gut microbiota, metabolism profiling

## Abstract

**Objectives:** To investigate the role of the intestinal flora and metabolites in the development of hyperuricemic renal injury in chronic kidney disease (CKD).**Methods:** Unilaterally nephrectomized mice were fed with adenine and potassium oxonate for 9 weeks. HE staining combined with plasma biochemical indicators was used to evaluate renal pathological and functional changes. We conducted 16S rRNA sequencing and untargeted metabolomics on feces and plasma samples to reveale changes in intestinal microbiota and metabolites.**Result:** Our analysis revealed significant differences in 15 bacterial genera, with 7 being upregulated and 8 being downregulated. Furthermore, metabolomic analysis revealed changes in the distribution of amino acid and biotin metabolites in basic metabolic pathways in both feces and serum. Specifically, differentially abundant metabolites in feces were associated primarily with histidine metabolism; the biosynthesis of phenylalanine, tyrosine, and tryptophan; and tyrosine metabolism. In plasma, the differentially abundant metabolites were involved in multiple metabolic pathways, including aminoacyl-tRNA biosynthesis; glycine, serine, and threonine amino acid metabolism; valine, leucine, and isoleucine biosynthesis; tyrosine biosynthesis and metabolism; biotin metabolism; and taurine and hypotaurine metabolism. Furthermore, correlation analysis revealed that *Akkermansia, UCG-005, Lachnospiraceae_NK4A136_group, Lactococcus, and Butymonas* were associated with various differentially abundant metabolites as well as renal function, oxidative stress, and mitophagy. The changes in the intestinal flora observed in hyperuricemia may lead to imbalances in amino acid and biotin metabolism in both the intestine and host, ultimately affecting oxidative stress and mitophagy in mice and accelerating the progression of CKD.**Conclusion: **Our findings provide insights into a potential pathogenic mechanism by which hyperuricemia exacerbates renal injury in mice with renal insufficiency. Understanding these pathways may offer new therapeutic strategies for managing hyperuricemic renal injury in CKD patients.

## Introduction

The incidence of hyperuricemia (HUA) has significantly increased worldwide, posing a new challenge to public health. Uric acid has been identified as a potential risk factor for the development and progression of chronic kidney disease (CKD) [1]. When the uric acid concentration exceeds the physiological range, various pathological responses can occur, including oxidative stress, mitochondrial dysfunction, apoptosis, and inflammation [2, 3]. One important mechanism by which hyperuricemia induces CKD is through mitochondrial dysfunction. The oxidant urate can increase the production of mitochondrial superoxide and excessive reactive oxygen species, causing damage to mitochondrial DNA and the electron transport chain. This ultimately results in renal tubular atrophy and nephron loss [4, 5]. This process is accompanied by the activation of autophagy in renal tubular epithelial cells. Studies have shown that hyperuricemia-induced autophagy is closely associated with the activation of renal fibroblasts, mitochondrial fission, the apoptosis of renal tubular epithelial cells, and the development of renal fibrosis [6]. According to current studies, an imbalance of the intestinal flora and damage to the intestinal mucosal barrier can also contribute to kidney damage. Alterations in the gut microbiota, as well as disturbances in the fecal and serum metabolome, are associated with the severity of chronic kidney disease [7]. Several studies have reported associations between CKD, hyperuricemia, and the intestinal microbiota [8–10]. For example, CKD patients show decreased levels of *Faecalibacterium, Roseburia, Cluster IV Clostridium, Eubacterium, Bifidobacterium, and Lactobacilliaceae,* along with increased colonization of *Enterobacteriaceae* [11].

Additionally, patients with hyperuricemia exhibit a decrease in the abundance of *Bacteroidetes* and *Proteobacteria*, whereas the abundance of *Firmicutes* and *Actinobacteria* increases [9]. Metabolomics is increasingly used to analyze various kidney diseases, such as diabetic nephropathy (DN), lupus nephritis, and immunoglobulin A nephropathy [12–15]. These findings suggest that the gut microbiota and its associated metabolites interact with the host kidney through the microbiota–gut–kidney axis, making them potential targets for early detection and personalized drug therapy to slow renal progression.

Therefore, in this study, we conducted gut 16S rRNA sequencing, as well as fecal and serum metabolome analyses, in a unilateral nephrectomy mouse model and administered adenine and potassium oxoacids to investigate microbial-related metabolites and metabolic pathways that may contribute to the underlying mechanism of injury.

## Methods and materials

Reagent Preparation The gastric infusion solution was prepared using 5% sodium carboxymethylcellulose (CMC-Na) as the solvent. To prepare the intragastric solution, we accurately weighed 3000 mg of potassium oxonate and 200 mg of adenine. Next, we added 30 mL of 0.5% CMC-Na to each mixture, resulting in a suspension with a concentration of 1500 mg/kg of potassium oxonate and 100 mg/kg of adenine.

### Experimental animals and study design

SPF-grade 8-week-old male C57 mice were obtained from Shanghai Slack Laboratory Animal Company and raised in a standard environment (average temperature of 22 °C with a standard 12-h/12-h light/dark cycle). After a week of adaptive feeding, a renal-insufficient mouse model was constructed *via* unilateral nephrectomy. We used the dorsal incision method to cross-fix the lower limbs of each mouse on the operating table. After anesthesia, the right kidney area on its back was exposed for disinfection. We selected an oblique outward incision 1.5 cm away from the right spine and ribs and cut it layer by layer. The abdominal wall structure reaches the abdominal cavity, and sterile surgical thread was used to ligate the renal hilum vessels. After the ligation was completed, the right kidney was removed, and the incision was sutured layer by layer. Sham operations (sham group, *n* = 6) were conducted at the same time points. After the model was successfully constructed, the mice were randomly divided into two experimental groups (*n* = 6 per group), (1) the UNx group, in which the mice were fed the same volume of CMC-Na as the control group by gavage once a day and (2) the UNx + HPD group, in which the mice were fed adenine (0.1 g/kg) and a suspension of potassium oxonate (1.5 g/kg) by gavage once a day.

**Figure 1.**
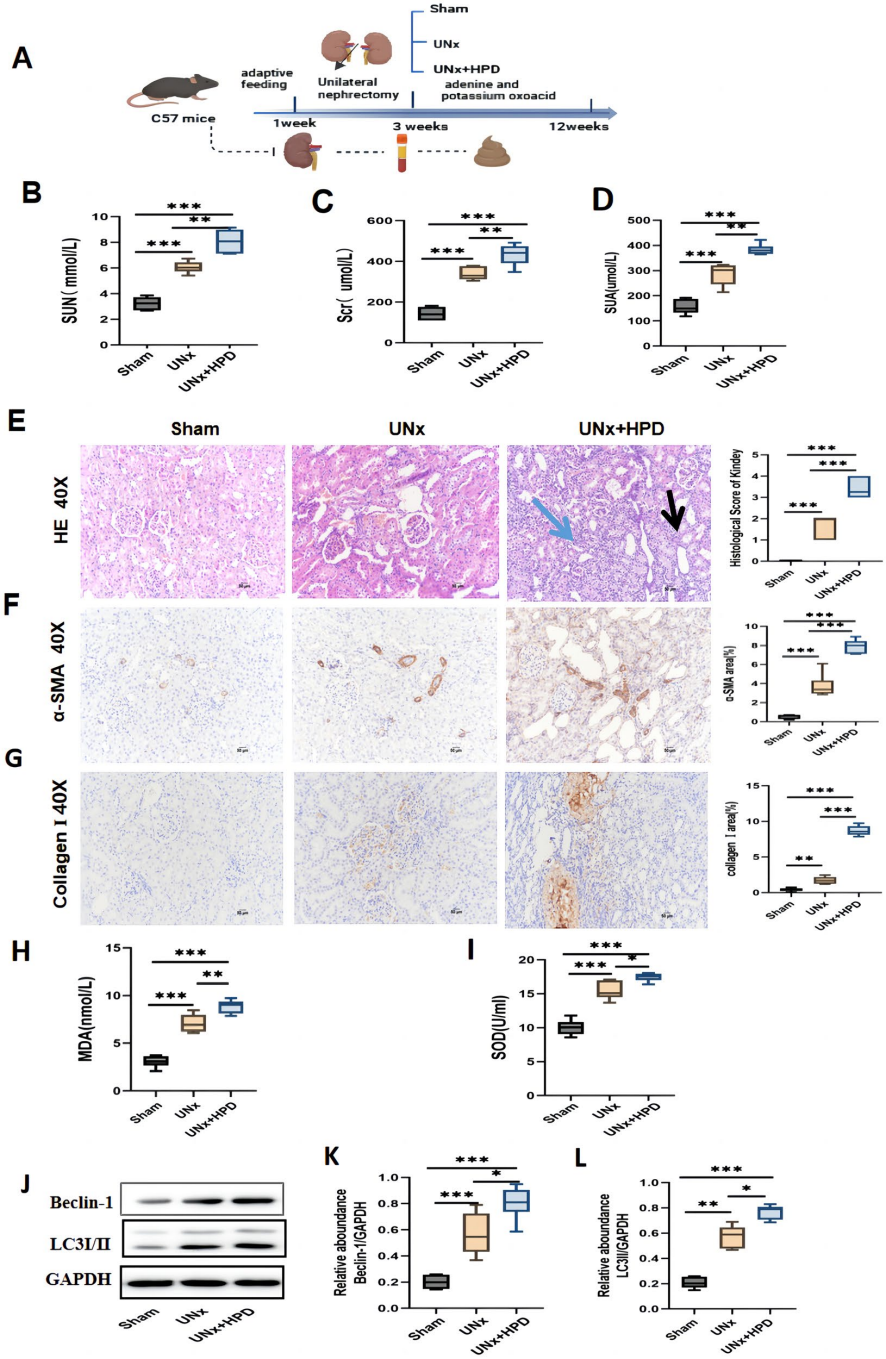
Kidney injury caused by hyperuricemia (HUA) in UNx mice. (A) The flowchart of the animal experiment. (B–D) Alteration of serum creatinine(scr), urea (SUN), and uric acid (SUA) in three groups of mice. The data are expressed as mean ± SEM (*n* = 6). (E) Representative images (40×) for hematoxylin and eosin(H&E) staining of kidney tissues and score of renal injury; green arrow points to renal interstitial inflammatory changes, the black arrow points to vacuolization and atrophy of the renal tubules. (F) Immunohistochemical staining of α-smooth muscle actin(α-SMA) in kidney tissues and percentage of α-SMA positive area. (G) immunohistochemical staining of collagen I in kidney tissues and percentage of collagen I positive area. (H-I) Concentrations of malondialdehyde(MDA) and superoxide dismutase(SOD) in kidney tissue; (J–L) Western blotting detection of expression of microtubule-associated protein 1 light chain 3 I/II(LC3I/II) and Beclin I in kidney tissue and histogram analysis. ns, not significant; *p < 0.05; **p < 0.01; ***p < 0.001. Analysis was performed by ANOVA followed by Tukey’s multiple comparison test. Sham, sham operations group; UNx, unilateral nephrectomy group; UNx + HPD, unilateral nephrectomy + adenine and potassium oxinate diet.

After nine weeks of feeding, blood was collected from the medial canthus vein. Finally, the mice were euthanized under deep anesthesia, and plasma, stool, and kidney samples were collected and stored at −80 °C for subsequent analysis. All experiments and operations on the mice were conducted in an SPF-grade animal breeding room. The experimental procedures were approved by the Animal Welfare and Ethics Group of the Department of Experimental Animal Science of Fudan University (Approval Number: 2021JS Minhang Hospital-012). The mouse treatment flow chart is shown in Figure 1(A).

### Biochemistry and ELISA

Serum creatinine (SCR) and the ratio of blood urea nitrogen (BUN) to serum uric acid (SUA) were measured using commercial kits. Malondialdehyde (MDA) and superoxide dismutase (SOD) were quantified using an ELISA kit.

### Histological and immunohistochemistry analysis

The kidneys were fixed in 10% neutral buffered formalin for 48 h, embedded in paraffin, and subjected to hematoxylin–eosin (HE) staining, and histopathological changes were observed *via* a light microscope (Olympus, Japan) to evaluate the pathological damage to the kidneys. The fixed tissue sections were dewaxed, rehydrated, quenched, and sealed. The sections were incubated overnight with appropriate primary antibodies (α-SMA and collagen I primary antibodies (1:200); Boster, Wuhan (China)) and secondary antibodies (Boster, Wuhan, China) at room temperature. After incubation for 1 h, the sections were stained with DAB reagent. Observations were then performed *via* an Olympus Provis AX70 microscope (Olympus, Japan). The data were quantitatively analyzed *via* Image-Pro Plus software.

### WB detection of autophagy indicators

Western blotting of kidney tissue was performed as previously described. The primary antibodies against LC3I/II (1:1000 dilution) and Beclin I (1:1000 dilution) were purchased from Cell Signaling Technology, and the primary antibody against GAPDH (1:1000 dilution) was purchased from Sigma–Aldrich.

### Sample preparation for intestinal flora analysis

Fresh feces were collected and transported on dry ice before the mice were sacrificed. The samples were then stored at −80 °C until DNA extraction. DNA was extracted using the CTAB/SDS method. The DNA concentration and purity were measured on a 1% agarose gel and diluted to 1 μg/μL with sterile water. The 16S rRNA in the V3-V4 region of all the ­samples was amplified using the primers 338 F (5′-actcctacgggaggcagcag-3′) and 806 R (5′-ggactachvggggtwtctaat-3′). The PCR products were purified using a gel extraction kit after being mixed at an isopycnic ratio. A high-throughput sequencing library was constructed on the Illumina platform and sequenced using the Illumina NovaSeq platform, generating 250 bp paired-end reads. The raw tags were merged and quality filtered using the FLASH (VI.2.7) platform to obtain high-quality sequences *via* QIIME (V1.9.1). Chimeras were removed using the UCHIME algorithm with the Silva database. Sequences with a similarity greater than 97% were classified into the same operational taxonomic units (OTUs) using UPARSE (V7.0.1001). The taxonomic information of the sequences was annotated based on the Mothur algorithm using the Silva database.

### Data processing

The α diversity and β diversity were calculated using QIIME (V1.7.0). Nonmetric multidimensional scaling (NMDS) analysis based on the Bray–Curtis distance was performed using the vegan package in R software (V2.15.3).

## Nontargeted metabolomics detection

### Sample preparation

The extraction solution was prepared using a 2:2:1 volume ratio of methanol, acetonitrile, and water. An isotope-labeled internal standard mixture was added. A 25-mg fecal sample was taken, and 500 μL of extract was added. The sample was centrifuged after grinding and placed in an ice-water bath. The supernatant was then tested on a machine. An equal amount of supernatant was taken from all the samples and mixed to create a quality control (QC) sample for testing on the machine. The target compounds were separated using a Waters ACQUITY UPLC BEH Amide LC column on an ultra-high-performance liquid chromatograph. A Thermo Q Exactive HFX mass spectrometer, controlled by Xcalibur software (version: 4.4, Thermo), was used to identify and collect primary and secondary mass spectrometry data.

### Mass spectrometry analysis

MetaboAnalyst (https://www.metaboanalyst.ca) was used for data cleaning, statistical analysis, and pathway enrichment analysis. Various preparations and adjustments were performed on the raw data to improve the biological relevance of the results and reduce the influence of detection system errors. This involved filtering out outliers and removing individual peaks that were considered noise. Deviations were filtered based on the relative standard deviation (RSD), also called the coefficient of variation (CV). Additionally, we performed missing value filtering on individual peaks, retaining only the peak area data with null values not exceeding 50% in a single group or across all groups. For missing value records, we replaced them in the original data with half of the minimum value.

Data normalization was performed using normalization with an internal standard (IS). For more than 50% of the samples, the peak intensity was removed from the peak intensity matrix. The remaining missing values were replaced with one-fifth of the smallest positive value for each variable. If the relative standard deviation (RSD) of the deviation was greater than 25%, filtering and normalization were performed using the mean. MetaboAnalyst (https://www.metaboanalyst.ca) was implemented for data cleaning, statistical analysis, and pathway enrichment analysis. The Orthogonal Projection Latent Structure Discriminant Analysis (OPLS-DA) algorithm, fold change (FC), and t test were used to identify metabolites with significant differences between groups. The OPLS-DA model was validated using permutation experiments (100 permutations). Differentially expressed metabolites (DEMs) were identified by strict criteria, namely, variable importance in the projection (VIP) value > 1, log2 (fc) > |2|, and *p* < 0.05. The Kyoto Encyclopedia of Genes and Genomes (KEGG) pathways of the DEMs were enriched using MetaboAnalyst and are presented as potential targets with a threshold of *p* < 0.05.

### Statistical analysis

Statistical tests were performed using GraphPad Prism software (V9.1.2) and R language (3.3.4). One-way ANOVA was used to analyze three or more groups of continuous data that were normally distributed. For continuous data that did not follow a normal distribution among the three groups, the Kruskal–Wallis H test was used. Unpaired Student’s t test used for parametric tests comparing two groups, whereas the Mann–Whitney U test was used for nonparametric data. Differential OTUs were identified using the DEseq2 package with thresholds of *p* < 0.05 and log2(fc) >|1|. Spearman correlations were used to analyze potential relationships between key DEMs and OTUs or parameters associated with CKD. Statistical Notes: ns, not significant; *p < 0.05; **p < 0.01; ***p < 0.001. The relevant graphics were generated using the R language platform.

## Results

### Biochemical indicators and renal pathological changes in the mice

The flowchart of the animal experiment is shown in Figure 1(A). Mice were randomly divided into three groups after adaptive feeding, and the total experimental duration was 12 weeks. Figure 1(B–D) shows that the levels of serum uric acid (SUA), creatinine (Scr), and urea (SUN) increased in the UNx and UNx + HPD groups, whereas the UNx + HPD group presented a more significant increasing trend than the UNx group. Moreover, the results of renal pathological changes were consistent with those of renal function changes. As shown in Figure 1(E), HE staining revealed the structures of the glomeruli and renal tubules in the kidney tissue of the sham mice were normal. In the UNx group, mild glomerular mesangial matrix hyperplasia and tubular vacuolization were observed, whereas the degree of renal tissue damage was significantly aggravated in the UNx + HPD group, with tubular vacuolation or atrophy, interstitial inflammatory infiltration and glomerulosclerosis. Immunohistochemical images of α-smooth muscle actin (α-SMA) and collagen I are shown in Figure 1(F). Positive areas of α-SMA and collagen I staining were visible in the images of the mice in the UNx group, and the positive staining of α-SMA and type collagen I was more obvious in the UNx + HPD group. These findings indicate that a high-purine diet can exacerbate kidney injury and lead to a decline in renal function in mice undergoing unilateral nephrectomy.

As mentioned previously, oxidative stress and mitophagy caused by hyperuricemia are the main mechanisms involved in renal injury. We detected malondialdehyde (MDA) and superoxide dismutase (SOD) in kidney tissues to evaluate the levels of oxidative stress (Figure 1H,I). Compared with those in the sham group, the levels of MDA and SOD increased in the UNx group; furthermore, the UNx + HPD group exhibited significantly elevated levels of MDA and SOD, suggesting a more severe oxidative stress response due to hyperuricemia in these mice. To assess changes in mitophagy in mice, we examined the expression of markers of autophagy, namely, microtubule-associated protein 1 light chain 3 (LC3) and Beclin I, which play crucial roles in the initial steps of autophagy. As shown in Figure 1(J–L), compared with those in the sham group, the expression of LC3I/II and Beclin-1 was significantly increased in the UNx (*p* < 0.05) and UNx + HPD groups (*p* < 0.05). The LC3I/II and Beclin-1 levels were greater in the UNx + HPD group than in the control group, suggesting that both unilateral nephrectomy and high uric acid levels can promote mitophagy in renal tissue.

### Alterations in the gut microbiota of UNx mice fed a high-purine diet based on 16S rRNA sequencing

**Figure 2.**
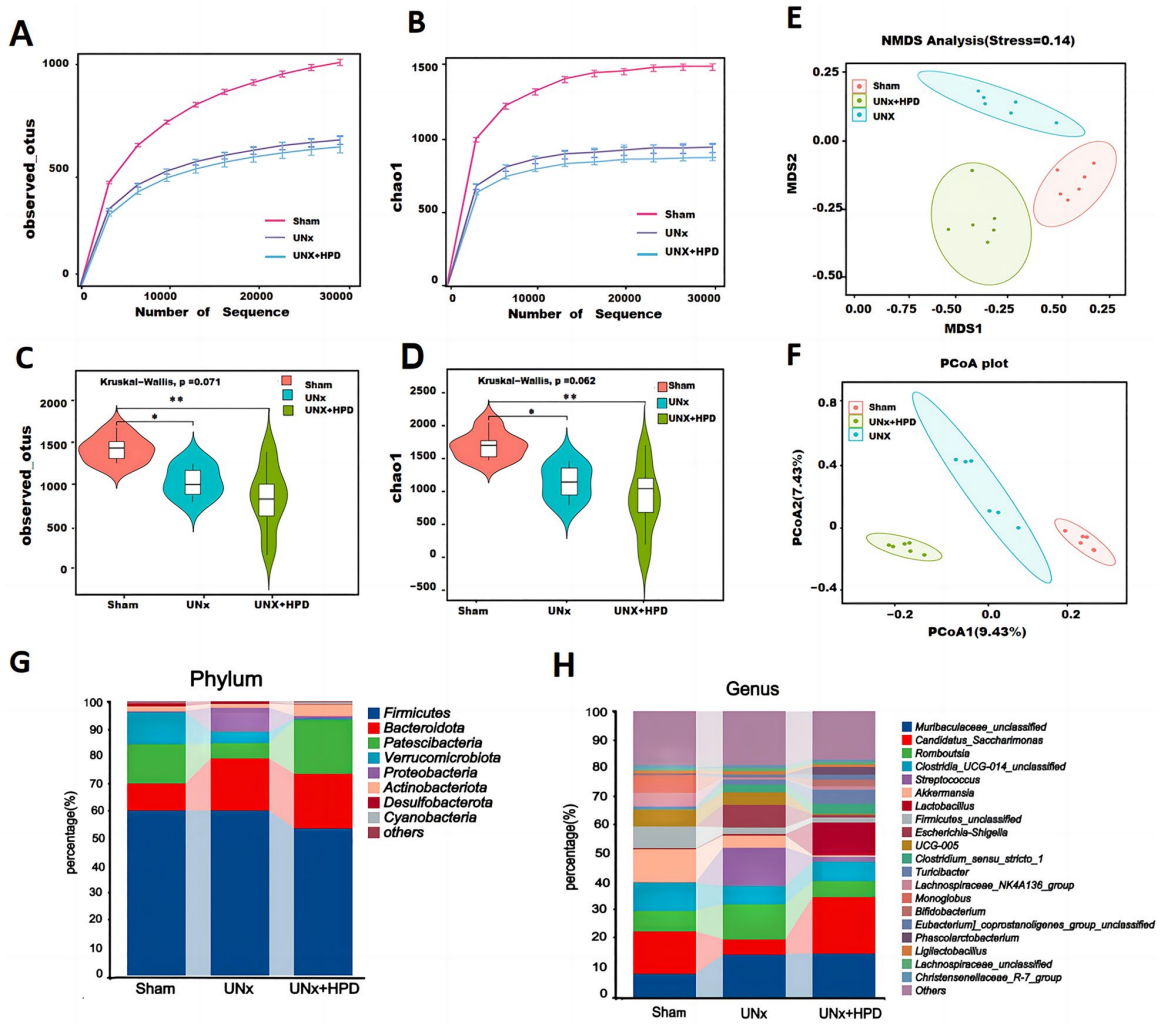
A high-purine diet altered the fecal microbiota of UNx mice. (A,B) Rarefaction curves showing different numbers of operational taxonomic units(OTUs) and Chao 1 index among three comparisons. (C,D) Violin plot showing different numbers of OTUs and Chao 1 index among three comparisons. (E, F) Principal coordinates analysis (PCoA) and non-metric multidimensional scaling(NMDS) showed significant differences in the intestinal flora of the three groups. (G) Stacked bar graph showing relative abundance of gut bacterial at phylum level. (H) Stacked bar graph showing relative abundance of gut bacteria at genus level. *p < 0.05, **p < 0.01. Sham, sham operations group; UNx, unilateral nephrectomy group; UNx + HPD, unilateral nephrectomy + adenine, and potassium oxinate diet.

Considering the influence of UNx and a high-purine diet on the gut microbiome, we explored the structure and differences in the gut microbiota of mice. The α diversity of the microbiome was estimated using two indices. As shown in Figure 2(A–D), fewer OTUs were observed in the UNx (*p* < 0.05) and UNx + HPD groups (*p* < 0.01) than in the sham group. The Chao1 indices of the UNx (*p* < 0.05) and UNx + HPD groups (*p* < 0.01) were significantly lower than that of the sham group. These findings suggest that UNx and HUA decreased α diversity; however, there was no significant difference in α diversity between the UNx and UNx + HPD groups. To further analyze the β diversity of the microbial composition, we performed NMDS analysis and PCoA (Figure 2E,F). The analyses revealed that the sham group was significantly separated from the UNx and UNx + HPD groups. The results suggest that a high-purine diet has a greater effect on the overall structure of the gut microbiota than UNx.

As shown in Figure 2(G), compared with that in the sham group, the Firmicutes population in the UNx + HPD group was decreased, whereas that in the UNx group was not significantly different. In contrast, the Bacteroides population in both the UNx and UNx + HPD groups was greater than that in the sham group. In addition, compared with that in the sham group, the *Verrucobacteria* population was decreased in the UNx and UNx + HPD groups. Compared with those in the sham and UNx groups, the *Proteobacteria* and *Actinobacteria* populations were significantly increased in the UNx + HPD group. At the genus level, *Muribaculaceae_unclassified, Candidatus_Saccharimonas, Romboutsia Clostridia_UCG-014_unclassified, Streptococcus, Akkermansia, Lactobacillus, Firmicutes_unclassified, Escherichia–Shigella, UCG-005, Clostridium_sensu_stricto_1, Turicibacter, Lachnospiraceae_NK4A136_group, Monoglobus, Bifidobacterium* were among the top 20 most abundant genera. The histogram shows the distribution of these genera in each group (Figure 2H).

**Figure 3.**
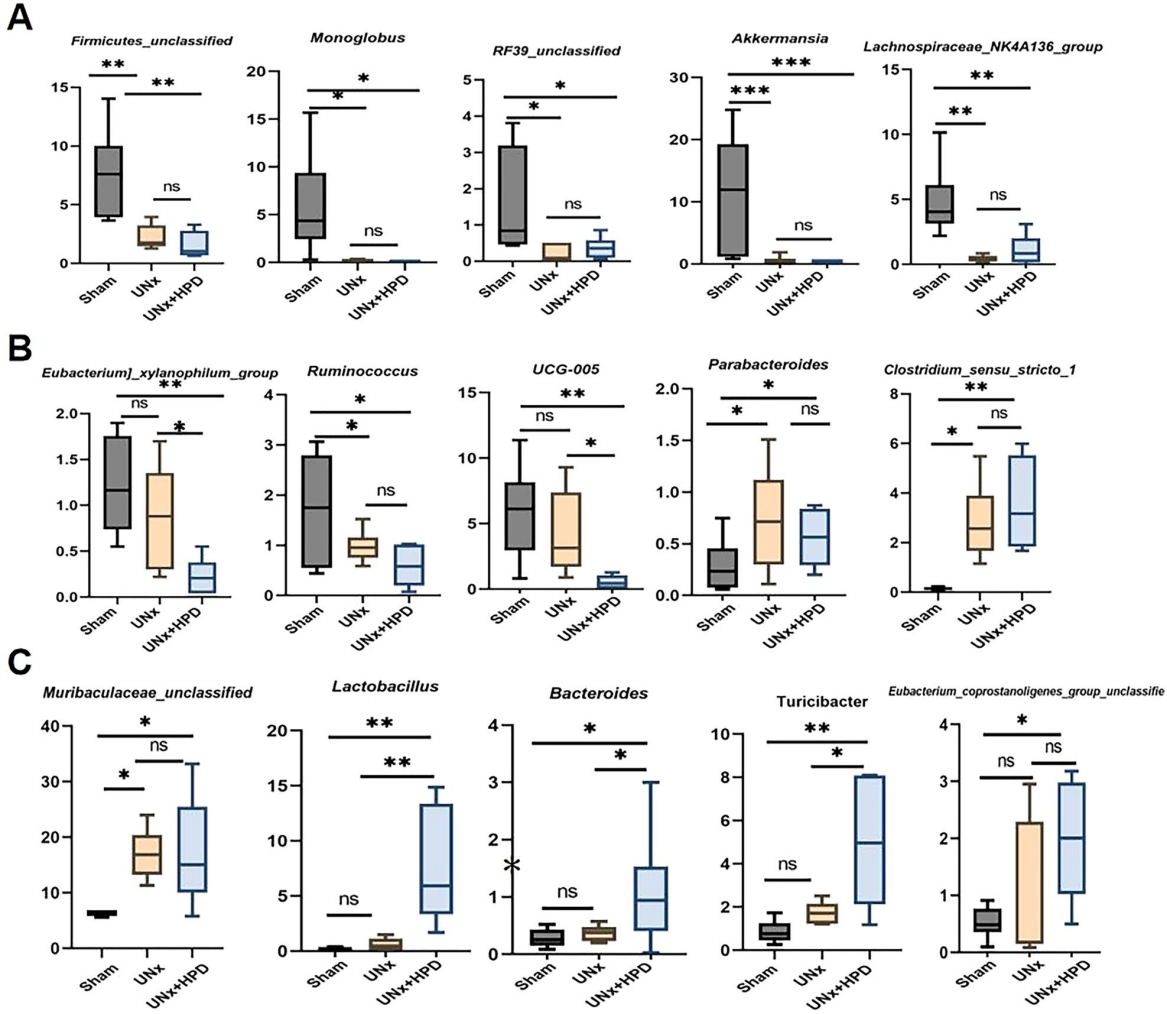
Identification of specific altered bacteria of UNx mice fed by a high-purine diet. (A–C) Changes in the intestinal flora of mice in the three groups (15 of the top 30) have statistical differences (ANOVA test). ns, not significant; *p < 0.05, **p < 0.01, ***p < 0.001. Sham, sham operations group; UNx, unilateral nephrectomy group; UNx + HPD, unilateral nephrectomy + adenine and potassium oxinate diet.

Next, we compared the changes in the 15 most abundant genera, as shown in Figure 3(A–C). Compared with those in the sham group, the abundances of *Firmicutes_unclassified, Monoglobus, Escherichia-Shigella, RF39 unclassified, Akkermansia, Lachnospiraceae, Lachnospiraceae_NK4A136_group*, and *Ruminococcus,* were reduced in both the UNx and UNx + HPD groups. In contrast, the abundances of *Parabacteroides, Muribaculaceae_unclassified,* and *Clostridium_sensu_stricto_1* were increased in both the UNx and UNx + HPD groups, whereas these genera did not differ between the groups. Compared with those in the sham and UNx groups, the abundances of *Bifidobacterium, Lactobacillus,* and *Turicibacter* were greater in the UNx + HPD group, and the abundances of *UCG-005* and *Eubacterium_xylanophilum_group* were lower in the UNx + HPD group.

### Fecal metabolomic profiling and correlation of differential bacteria with metabolites

**Figure 4.**
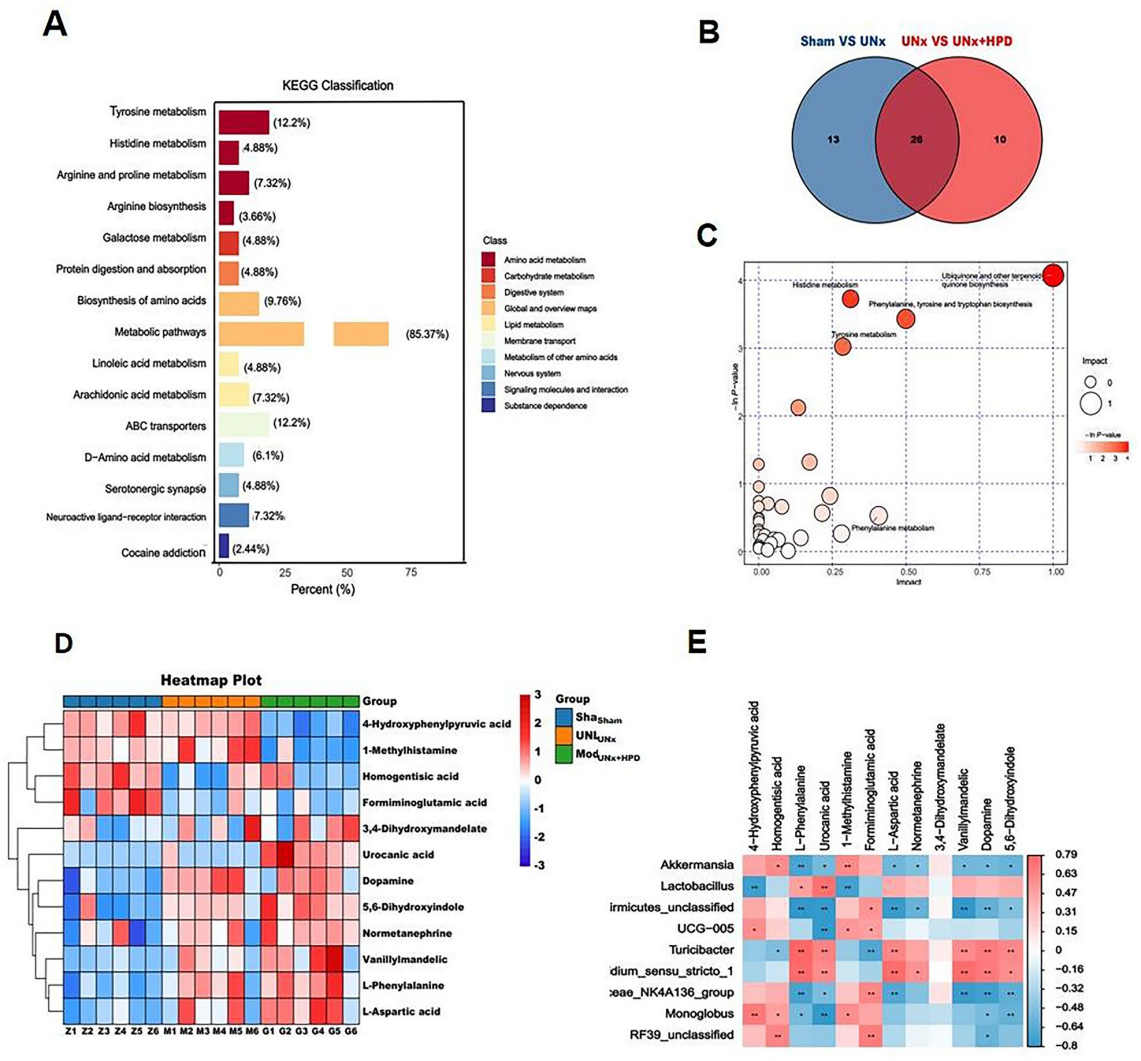
Altertion of metabolomic profiling in fence. (A) Kyoto encyclopedia of genes and genomes (KEGG) classification of fecal differential metabolites between Sham group and model group; (B) Venn plot showing the differential fecal metabolites between Sham group and UNx group, UNx group and UNx + HPD group; (C) Pathway analysis of differential metabolites in feces of Sham group and UNx + HPD group; (D) Heatmap analysis of metabolites involved in differential metabolic pathways of three groups; (F) Correlation analysis of differential metabolites and differential bacteria at genus level. Sham, sham operations group; UNx, unilateral nephrectomy group; UNx + HPD, unilateral nephrectomy + adenine and potassium oxinate diet.

LC–MS metabolic profiling results revealed that differentially abundant fecal metabolites were analyzed using the OPLS-DA model (VIP ≥ 1.0, fold change ≥ 2 or less than or equal to 0.5, and P less than 0.05). A total of 340 differentially abundant metabolites were identified, with 175 metabolites upregulated and 165 metabolites downregulated. The distribution of these differentially abundant metabolites was analyzed using a volcano plot (Supplementary Figure 1). Further KEGG enrichment analysis of the differentially abundant metabolites revealed that they were associated primarily with amino acid metabolism, including tyrosine metabolism (12.2%), arginine and proline metabolism (7.32%), and histidine metabolism (3.66%). Additionally, carbohydrate metabolism, specifically galactose metabolism (4.88%), and protein digestion and absorption (4.88%), were also identified as significant metabolic pathways (Figure 4A).

Metabolic pathway enrichment analysis was performed on the KEGG database to explore the functional significance of these DEMs. Differentially abundant metabolite pathway analysis revealed 39 differential metabolic pathways between the sham and UNx groups, as well as 36 differential metabolic pathways between the UNx and UNx + HPD groups. Additionally, 16 differentially abundant metabolites were identified in both groups (Figure 4B). Pathway analysis revealed that the differentially abundant metabolites in the sham and UNx + HPD groups were involved primarily in the biosynthesis of ubiquinone and other terpenoid-quinones; histidine metabolism; phenylalanine, tyrosine and tryptophan biosynthesis; and tyrosine metabolism (Figure 4C). Heatmap analysis of differentially abundant metabolites involved in pathways revealed significant reductions in 4-hydroxyphenylpyruvic acid, 1-methylhistamine, homogentisic acid, and formiminoglutamic acid when the UNx + HPD group was compared with the sham group; conversely, an increasing trend was observed in L-phenylalanine, urocanic acid, L-aspartic acid, normetanephrine, 3,4-dihydroxymandelate, vanillymandelic, and dopamine5,6-dihydroxyindol (Figure 4D). Fifteen bacteria were selected for Spearman correlation analysis based on their significant differences and differentially abundant metabolites in the pathway. Among these genera, *Firmicutes-unclassified, UCG-005, Lachnospiridae_NK4A136_group, Lactococcus,* and *Butyromonas* showed positive correlations with 4-hydroxyphenylpyruvate, homogentisic acid, 1-methylhistamine, formiminoglutamic acid, L-aspartic acid, and norepinephrine. However, they are inversely related to the dopamine 5,6-dihydroxyindole vanillylmandelic acid (Figure 4E).

### Metabolomic profiling of plasma and correlation of differential bacteria with metabolites

**Figure 5.**
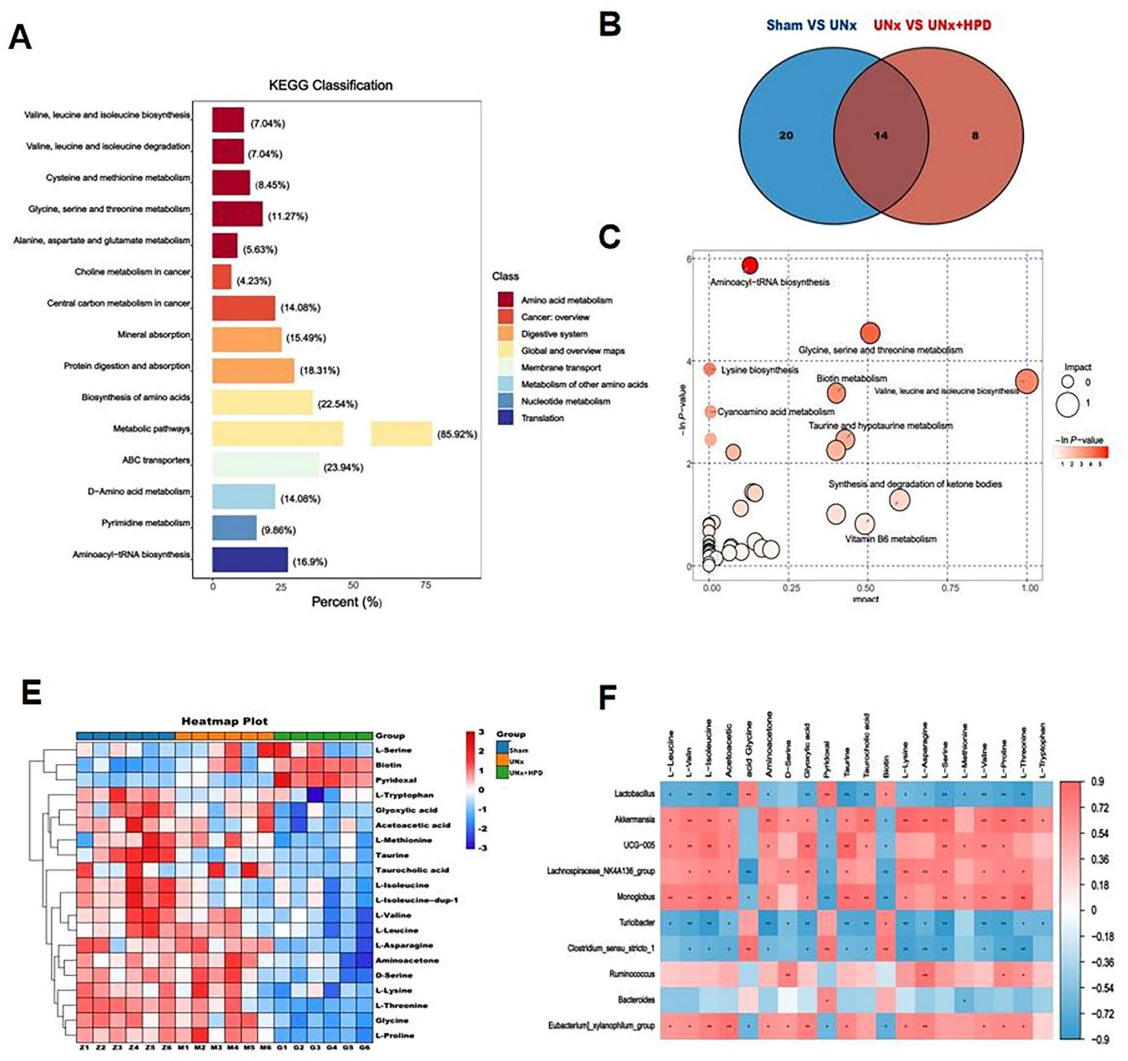
Change of metabolomic profiling in plasma. (A) KEGG classification of fecal differential metabolites between Sham group and model group; (B) Venn plot showing the differential plasma metabolites between Sham group and UNx group, UNx group and UNx + HPD group; (C) Pathway analysis of differential metabolites in feces of Sham group and UNx + HPD group; (D) Heatmap analysis of metabolites involved in differential metabolic pathways of three groups; (F) Correlation analysis of differential metabolites and differential bacteria at genus level. Sham, sham operations group; UNx, unilateral nephrectomy group; UNx + HPD, unilateral nephrectomy + adenine and potassium oxinate diet.

Through nontargeted metabolomic analysis of plasma samples, a total of 161 different metabolites were identified in the sham group compared with those in the UNx + HPD group. Among these metabolites, 51 were upregulated, and 110 were downregulated. The differentially abundant metabolite distribution was analyzed using a volcano plot (Supplementary Figure 2). Further KEGG enrichment analysis of the differentially abundant metabolites revealed significant associations with amino acid metabolism, specifically valine, leucine, and isoleucine biosynthesis (7.04%); valine, leucine, and isoleucine degradation (7.04%); glycine, serine, and threonine metabolism (11.27%); cysteine and methionine metabolism (8.45%); pyrimidine metabolism (9.86%); and other related pathways (Figure 5A). Differentially abundant metabolite pathway analysis revealed 34 differential metabolic pathways between the sham and UNx groups. Additionally, 22 differential metabolic pathways were observed between the UNx and UNx + HPD groups. Furthermore, the two groups exhibited 14 differentially abundant metabolites (Figure 5B). The differentially abundant metabolites were primarily associated with aminoacyl tRNA biosynthesis; glycine, serine, and threonine metabolism; metabolic pathways involving valine, leucine, acid, and tyrosine biosynthesis metabolism; biotin metabolism; and taurine and hypotaurine metabolism (Figure 5C).

To further investigate the potential effects of differential gut microbiota and plasma metabolites, we conducted Spearman analysis to establish a link between differentially abundant metabolites and the gut microbiota. The heatmap analysis of differentially abundant metabolites involved in the pathway revealed that, compared with those in the sham operation group, there was a downward trend in L-asparagine, glycine, L-serine, L-methionine, L-valine, L-lysine, L-isoleucine, L-leucine, L-threonine, L-tryptophan, L-proline, aminoacetone, D-serine, glyoxylic acid, L-isoleucine, taurine, taurocholic acid, and acetoacetic acid, whereas biotin and pyridoxal significantly increased (Figure 5D). Additionally, Spearman correlation analysis was used to analyze the differentially abundant metabolites of plasma metabolic pathways associated with 15 different bacteria. The results indicated that *Akkermansia, UCG-005, Monoglobus,* and *Lachnospiraceae_NK4A136_group* were positively related to L-leucine, L-valine, L-isoleucine, aminoacetone, D-serine, glyoxylic acid, taurine, taurocholic acid, L-lysine, L-serine, L-methionine, L-proline, and L-threonine. *Lactococcus* and *Turicibacter* were negatively related to these differentially abundant metabolites (Figure 5E).

According to the results of the plasma metabolic pathway analysis shown in Supplementary Table 1, certain amino acids, such as L-serine, L-methionine, L-lysine, L-proline, and L-threonine, are involved in aminoacyl-tRNA biosynthesis. Additionally, L-isoleucine is involved in valine, leucine and isoleucine biosynthesis, whereas aminoacetone and D-serine are involved in glycine, serine and threonine metabolism. Taurine and taurocholic acid are also involved in taurine and hypotaurine metabolism. Therefore, *Akkermansia, UCG-005, Monoglobus,* and *Lachnospiraceae_NK4A136_group* were positively associated with aminoacyl-tRNA biosynthesis; valine, leucine and isoleucine biosynthesis; glycine, serine and threonine metabolism; and taurine and hypotaurine metabolism, whereas *Lactococcus* and *Turicibacter* were negatively related to these metabolic pathways.

### Integrative analysis of the gut microbiota and metabolic profiling with biochemical indicators

To investigate the correlation between differentially abundant metabolites in plasma, feces, and flora, as well as indicators such as renal function, oxidative stress, and mitophagy, in the mice from each group, we performed Spearman correlation analysis.

**Figure 6.**
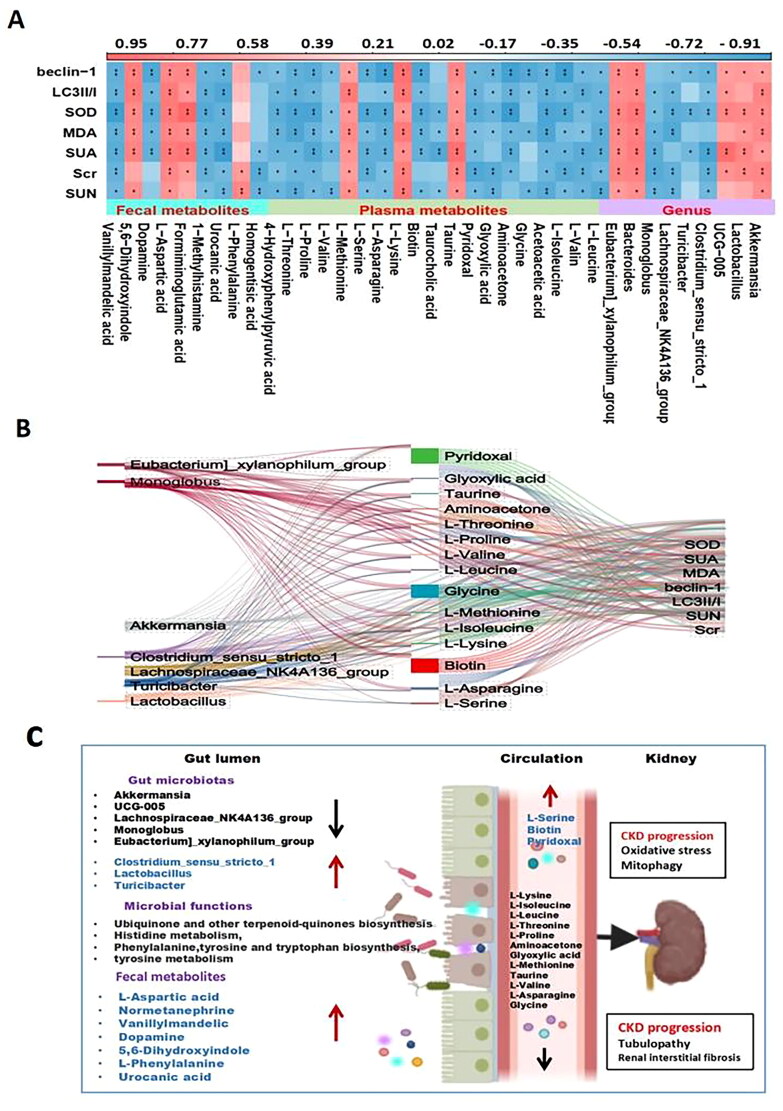
Correlation analysis of intestinal flora, metabolites and biochemical indicators. (A) Correlation analysis of differential flora, fecal metabolites, plasma metabolites and renal function among the sham group and UNx + HPD group; (B) Sanji diagram showing differences the relationship between the flora and plasma metabolites and biochemical indicators; (C) proposed mechanism of perturbed gut microbiome together with fecal and serum metabolites in pathogenesis of HUA induced-CKD progression. Red arrow indicates upregulation; black arrow indicates downregulation.

The relationship heatmap is shown in Figure 6(A). *Akkermansia, Lactobacillus, UCG-005, Bacteroides,* and *Eubacteria_xylanophilum_group* were positively correlated with these indicators. *Monoglobus* and *Turicibacter* were negatively correlated with each indicator. Additionally, some metabolites, such as 5,6-dihydroxyindole, formiminoglutamic acid, and L-aspartic acid, were positively correlated with the indicators, whereas L-methionine, urocanic acid, and dopamine were negatively correlated. Moreover, plasma metabolites such as biotin, L-serine, and pyridoxal were positively correlated with the related indicators, whereas L-leucine, L-isoleucine, acetoacetic acid, aminoacetone, D-serine, glyoxylic acid, taurocholic acid, L-lysine, L-asparagine, L-methionine, L-valine, L-proline, and L-threonine were negatively correlated. Further analysis involved selecting the differentially abundant metabolites and bacteria with R values greater than 0.7 and analyzing the Sanji diagram of each indicator (Figure 6B). The proposed mechanism of the role of the perturbed gut microbiome and fecal and serum metabolites in the pathogenesis of HUA-induced CKD progression is shown in Figure 6(C).

## Discussion

Recent studies have reported that intestinal microbial dysbiosis is a key factor in the pathophysiology of CKD, making it a new target for early diagnosis and personalized treatment [16]. However, the functional potential of hyperuricemia on the CKD gut microbiome and its complex interactions with metabolism remain poorly understood. Therefore, we conducted a comprehensive study investigating the effects of a high-purine diet on renal function in UNx mice. We found that the gut microbiome and fecal and serum metabolome of UNx + HPD mice were disrupted, which may mediate the destruction of toxic and oxidative stress metabolites, thereby accelerating the progression of CKD.

Here, we observed the diversity of the intestinal microbiota in UNx mice and UNx + HPD mice. Specifically, the relative abundances of Firmicutes and Proteobacteria decreased at the phylum level, whereas the relative abundances of Bacteroidetes, Proteobacteria, and Actinobacteria increased. An imbalance of the intestinal flora has been observed in patients with hyperuricemia and in animals, but the results obtained from different models are not only consistent. Zhang et al. reported that the richness and diversity of the microbiota in patients with hyperuricemia were reduced and that the relative abundance of Faecalicoccus was low [17]. In yeast and purine diet-induced HUA rat models, the relative abundances of Prevotella, anaerobic Vibrio, Alloprevotella, and Bartonella species were lower than those in control rats, whereas Allobaculum, Clostridium_XlVa, Flavonifractor, Phascolarctobacter, Clostridium_XVIII, Parabacteroides, Robinsoniella, Subdolicapsulum, Catabacter, Blautia, Bacteroides, and Olsenella were relatively more abundant than those in the control group [18]. In another study, in a purine diet-induced hyperuricemic nephropathy rat model, opportunistic pathogenic bacteria such as Flavobacterium, Myroides, Corynebacterium, Alcaligenaceae, and Oligella increased significantly, whereas the short-chain fatty acid (SCFA)-producing bacteria Blautia and Roseburia increased [19]. These changes in the microbiota suggest that gut microbial transformation may play a role in HUA. Notably, our experiments revealed that the abundances of *Bifidobacterium* and *Lactococcus* were significantly increased in the UNx + HPD group. Both of these are beneficial bacteria in the conventional sense. Lactobacilli can synthesize uricase, which can sequentially degrade UA into 5-hydroxyisosonic acid, allantoin, and allantoic acid and finally generate urea [20]. An increase in beneficial bacteria has also been reported in patients with asymptomatic hyperuricemia [21]. Whether this compensatory mechanism in the body still deserves further exploration.

Fecal metabolomics-based KEGG pathway analysis revealed that gut dysbiosis in the UNx + HPD group was related to the biosynthesis of ubiquinone and other terpenoid-quinones, histidine metabolism, phenylalanine, tyrosine, tryptophan biosynthesis, and tyrosine metabolism, and these pathways are associated with the inflammatory response and oxidative stress [22, 23]. Tryptophan is a nutritionally essential amino acid, and the TRP metabolic pathway usually consists of the kynurenine, serotonin, and indole pathways. The abnormal metabolism of TRP and its pathways is related to CKD [24]. Previous studies have emphasized the importance of tyrosine metabolism in renal function, as the production of renin, a hormone involved in renal processes such as water and salt metabolism and blood pressure regulation, is affected by tyrosine metabolism [25]. A study analyzing metabolome changes in long-lived *Drosophila melanogaster* during aging revealed that tyrosine levels increase with age and that tyrosine supplementation can significantly extend the lifespan of *Drosophila melanogaster*, which is related to the tyrosine degradation pathway. Downregulation of the rate-limiting enzyme tyrosine aminotransferase can induce metabolic reprogramming in Drosophila by affecting mitochondrial metabolism and antioxidant responses, ultimately delaying aging [26]. In this study, Spearman correlation analysis revealed positive results. A positive correlation was demonstrated between acetoacetate (part of the tyrosine metabolic pathway) and probiotics such as AKK. However, a negative correlation was found between oxidative stress indicators (MDA and SOD) and mitophagy indicators. These findings suggest that reduced tyrosine metabolism may be involved in renal impairment caused by hyperuricemia.

In untargeted metabolomics analysis of serum samples, several differentially abundant metabolites were related to several metabolic pathways, such as aminoacyl-tRNA biosynthesis; glycine, serine and threonine metabolism; valine, leucine acid, and isoleucine biosynthesis; tyrosine biosynthesis metabolism; biotin metabolism; and taurine and hypotaurine metabolism (Figure 5C). Multiple studies have shown that amino acid metabolism is highly related to hyperuricemia and gout. Studies have revealed positive correlations between alanine, isoleucine, leucine, phenylalanine, tryptophan, and valine levels and gout. In contrast, the levels of glycine and serine are negatively correlated with gout [27, 28]. In addition, the upregulation of valine, leucine, and isoleucine degradation metabolism can activate the mTOR signaling pathway, increase protein synthesis, and provide renal cells with energy, thereby improving renal function [27]. Therefore, further research and validation are needed to determine whether leucine and isoleucine metabolism in this model regulates renal cell metabolism through the mTOR signaling pathway.

Serine is generally considered a nonessential amino acid for humans; however, it plays a vital role in various cellular processes from a metabolic perspective, and its importance has been increasingly recognized in recent years [29, 30]. The kidney is the main site for serine production, and one of the main pathways involves the conversion of glycine, making glycine the main precursor for serine synthesis [31]. Glycine is the most abundant amino acid in the body and is involved in various metabolic pathways and biological processes. Under normal circumstances, the kidneys absorb glycine and release serine [32]. Hyperuricemic nephropathy may affect the conversion of glycine to serine in the kidney. In addition, renal and liver fibrosis are associated with serine, glycine, and hydroxyproline acid disorders and are related to metabolism [33]. In mice with uric acid nephropathy, the levels of serine, glutamate, and glutamine are reduced, whereas the glycine, hydroxyproline, and alanine levels are increased [19]. The abnormalities in serine and glycine metabolism found in this study are consistent with those reported in previous studies, suggesting that serine and glycine metabolism play a certain role in hyperuricemic renal injury.

Biotin plays a crucial role in the energy production process of cells by activating carboxylase. It also significantly affects the metabolism of carbohydrates, lipids, and proteins. A study conducted on Uox-Ko mice with uric acid nephropathy demonstrated disturbances in purine metabolism, amino acid biosynthesis, tryptophan metabolism, and neuroactive ligand–receptor interactions. Biotin is closely related to renal function and can potentially be a predictive plasma metabolic biomarker for urate nephropathy [33]. Our results showed that biotin metabolism was improved in the UNx + HPD group. Correlation analysis revealed that biotin metabolism positively correlates with renal function, oxidative stress, and other indicators. Our comprehensive analysis of the gut microbiota and metabolites revealed specific associations. *Akkermansia*, *UCG-005*, *Lachnospiridae_NK4A136_group*, *Lactococcus*, and *Butyromonas* were found to be associated with L-leucine, L-valine, L-isoleucine, aminoacetone, D-serine, glyoxylic acid, taurine, taurocholic acid, L-lysine, L-asparagine, L-serine, L-formazine thionine, L-proline, and L-threonine. These associations suggest that the decreased levels of these metabolites in UNx + HPD mice may be attributed to the downregulation of the flora.

Our study has several limitations. First, the sample size of each group was small. Expanding the sample size could lead to more reliable results. Second, the current research is limited to correlation studies. This study did not identify the specific bacterial types that play a major role in hyperuricemia-induced renal injury or how these bacteria modulate the identified differentially abundant metabolites to influence the progression of CKD. Therefore, further animal models and *in vitro* experiments are necessary to verify the role of the identified differential bacteria during hyperuricemia-induced renal injury.

## Conclusion

In renal injury induced by hyperuricemia in a renal-insufficient mouse model, the intestinal flora changed significantly, accompanied by differentially abundant metabolites in feces associated mainly with histidine, phenylalanine, tyrosine, and carbohydrate metabolism. The functional changes in the amino acid contrast pathway, whereas the differentially abundant metabolites in plasma are related mainly to aminoacyl-tRNA biosynthesis; glycine, serine and threonine metabolism; valine, leucine, and isoleucine biosynthesis; and tyrosine biosynthesis related to metabolic pathways such as anabolism and biotin metabolism. Furthermore, complex interactions among several CKD-related species and metabolites of four basic metabolic pathways are closely related to CKD progression. In addition, correlation analysis revealed that *Akkermansia*, *UCG-005*, *Lachnospiraceae_NK4A136_group*, *Lactococcus,* and *Butymonas* are related to a variety of differentially abundant metabolites and renal function, oxidative stress and mitophagy. Our multiomics analysis suggests the underlying mechanism of the microbiota–gut–kidney axis in the progression of early CKD with hyperuricemia and provides promising evidence for new targets for intervention in uric acid renal damage in the context of CKD.

## Supplementary Material

Figure 4.tif

Figure 3.tif

Supplementary_Material.doc

Figure 6.tif

Figure 5.tif

## Data Availability

The 16S rDNA sequencing data are available in the SRA of NCBI, accession number PRJNA1014247.
