# Supplementary figures and images for: Alterations in the gut microbiome and metabolism profiles reveal the possible molecular mechanism of renal injury induced by hyperuricemia in a mouse model of renal insufficiency
Source: Ren Fail. 2024 Aug 12;46(2):2387429. doi: 10.1080/0886022X.2024.2387429 (PMC11321104; doi:10.1080/0886022X.2024.2387429)

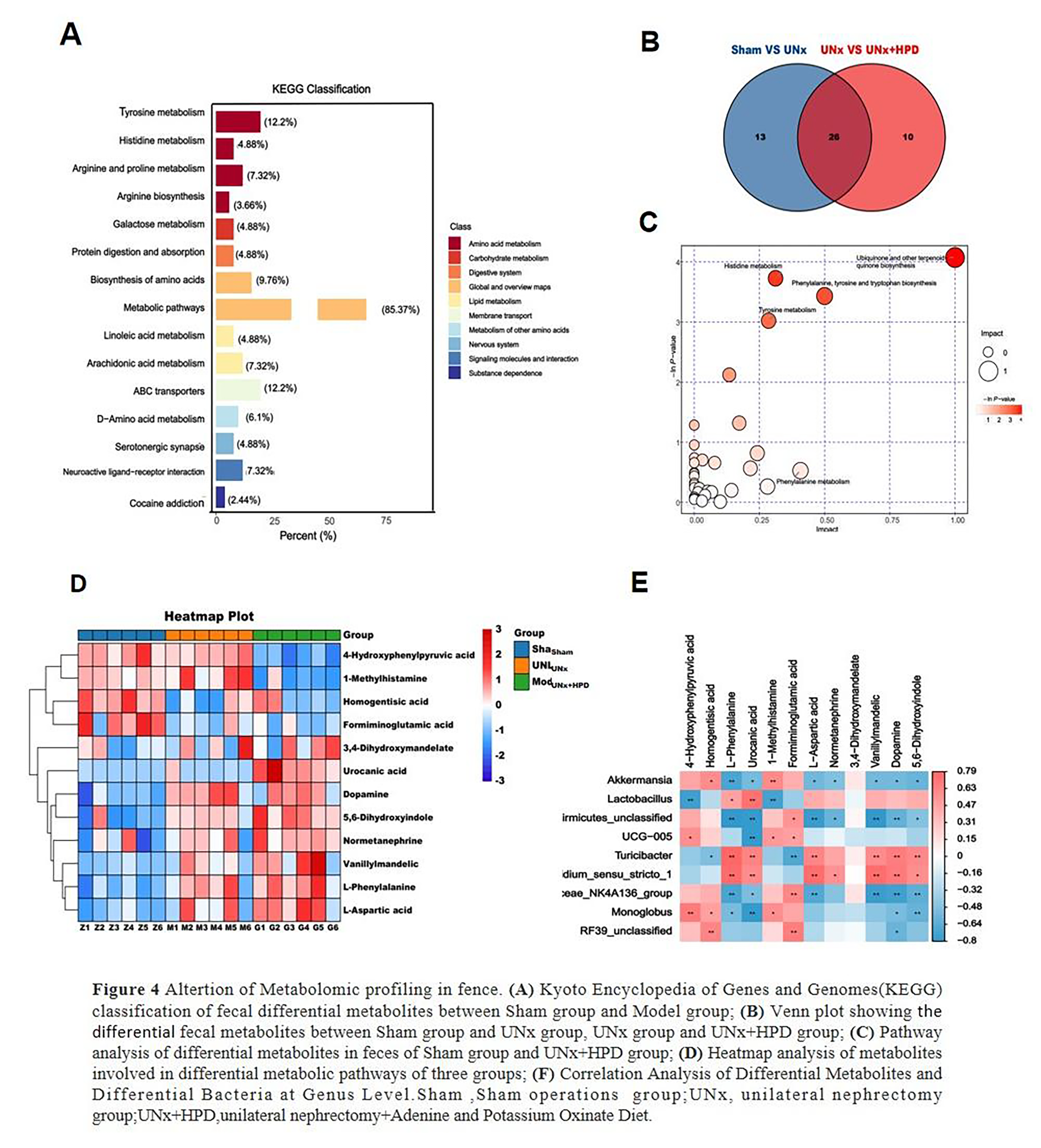

Supplement: Figure 4.tif [file IRNF_A_2387429_SM4136.tif]

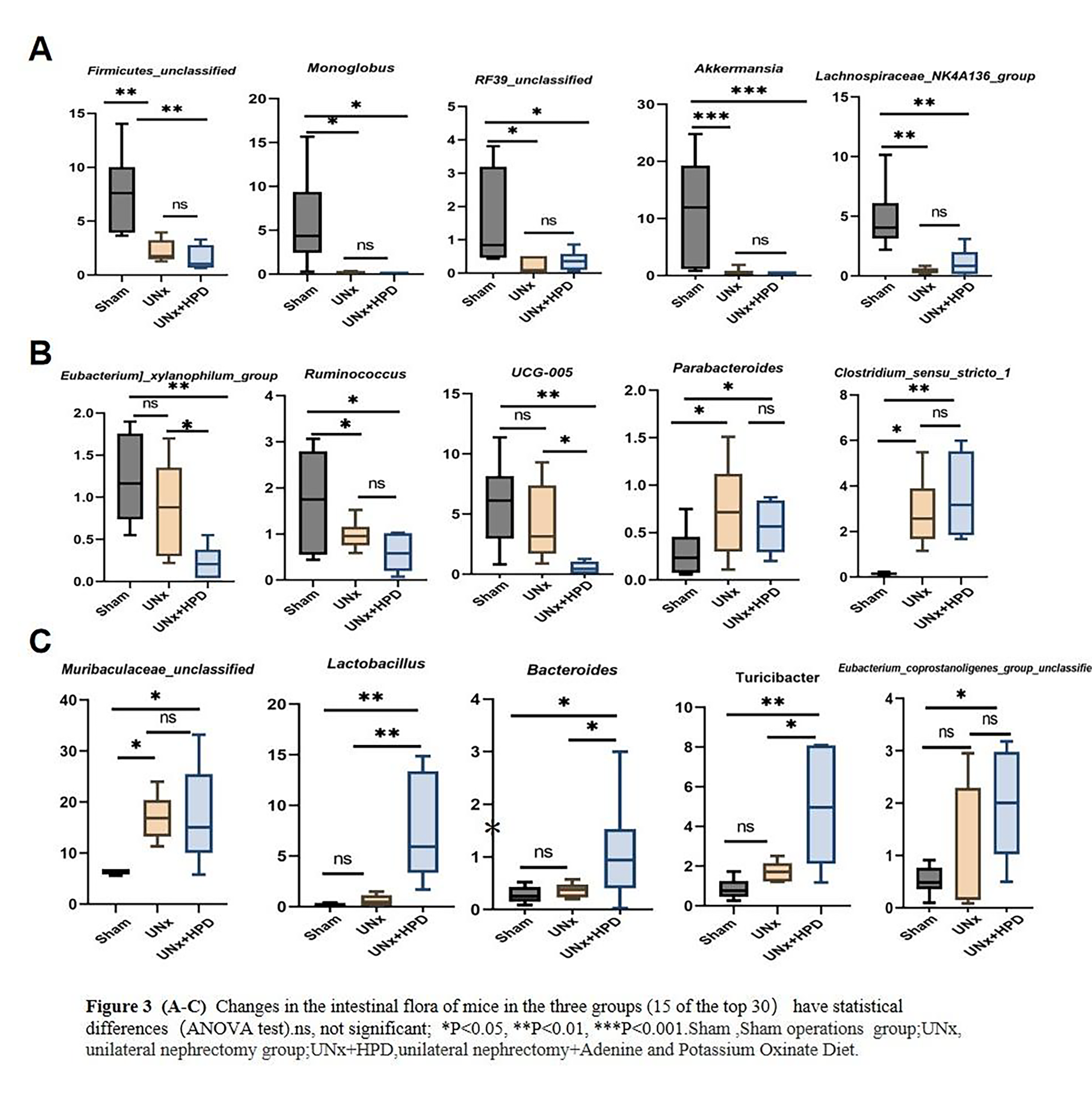

Supplement: Figure 3.tif [file IRNF_A_2387429_SM4135.tif]

Supplementary Material

# Supplementary Figures


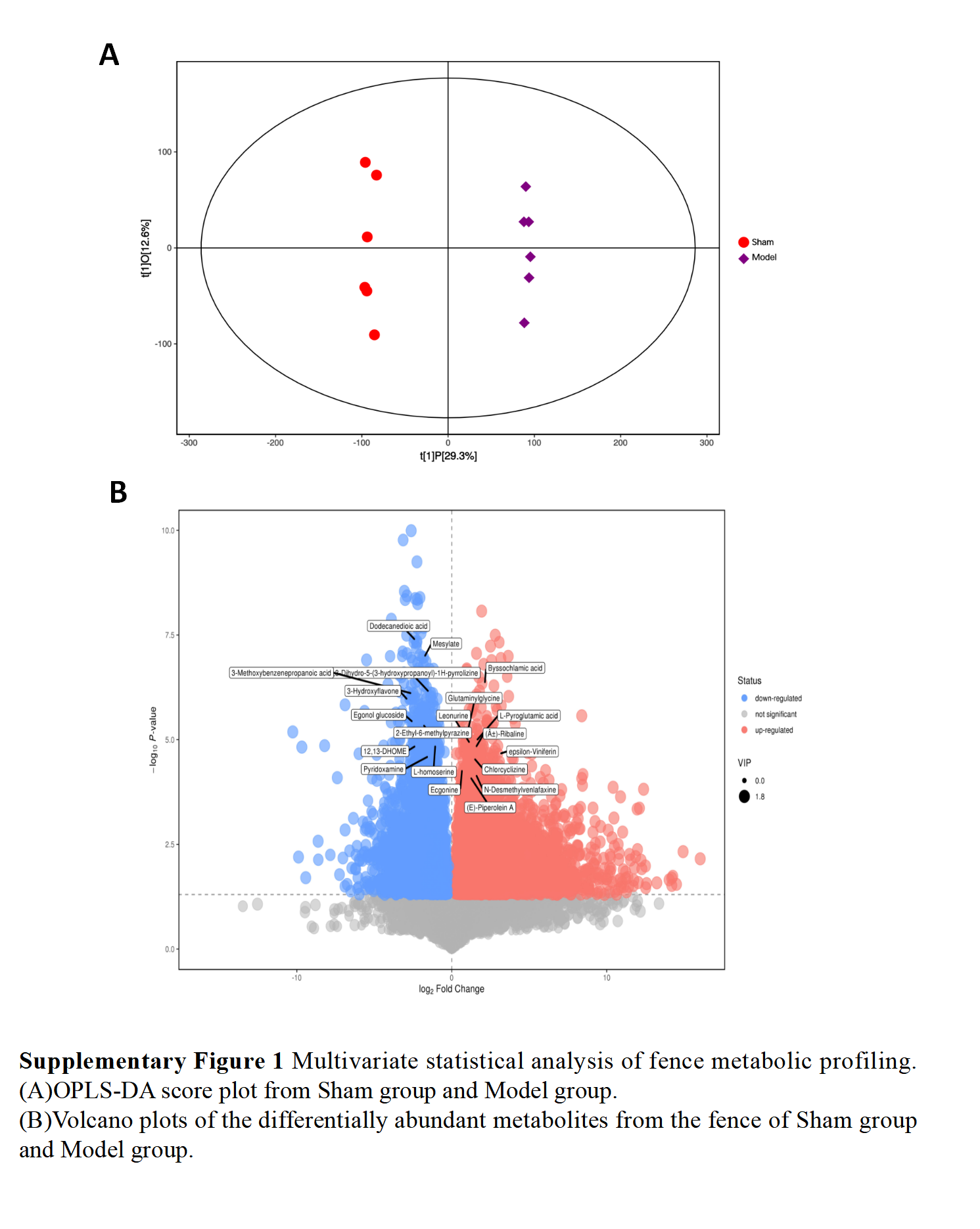


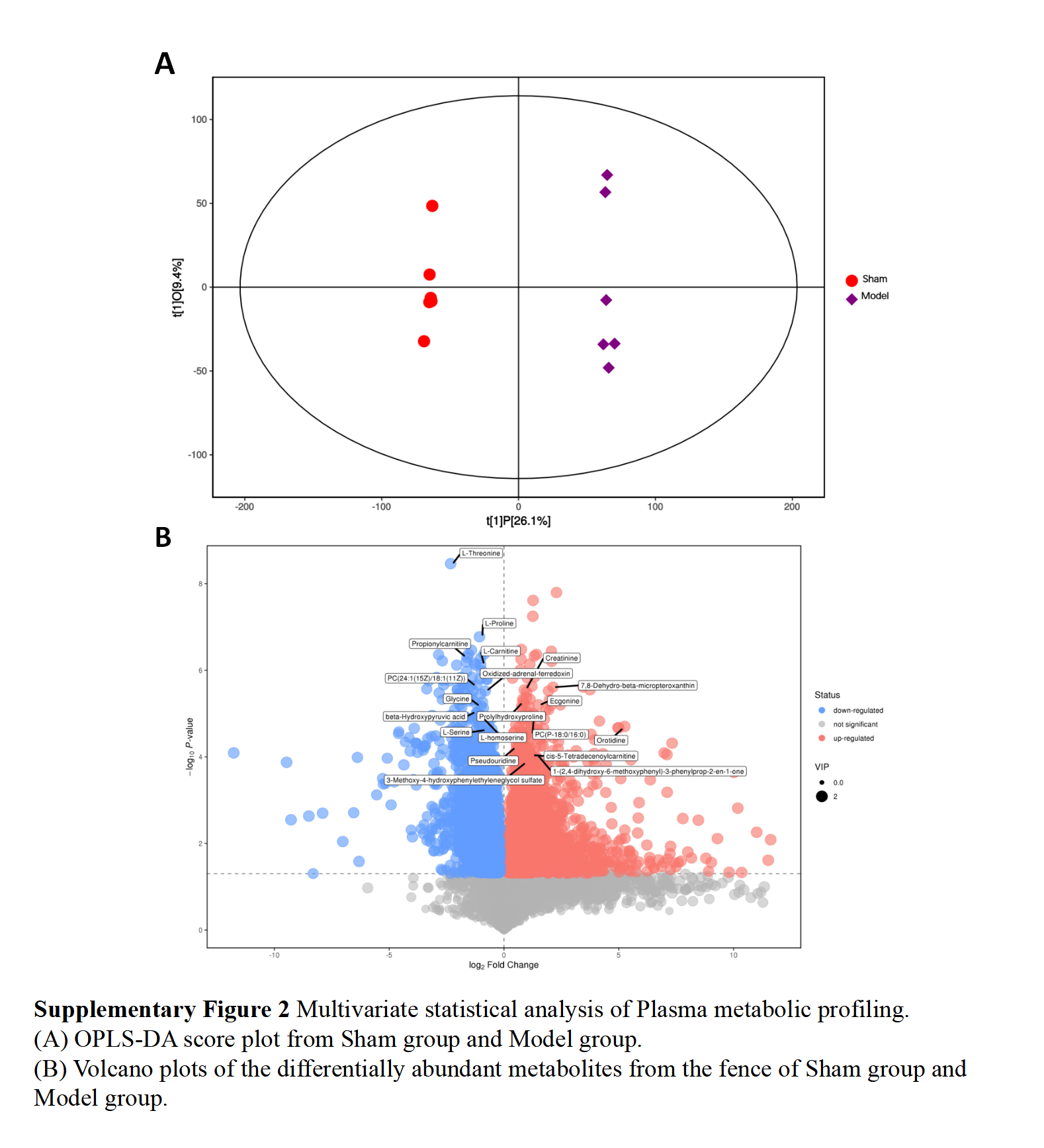

Supplement: Supplementary_Material.doc [file IRNF_A_2387429_SM4134.doc]

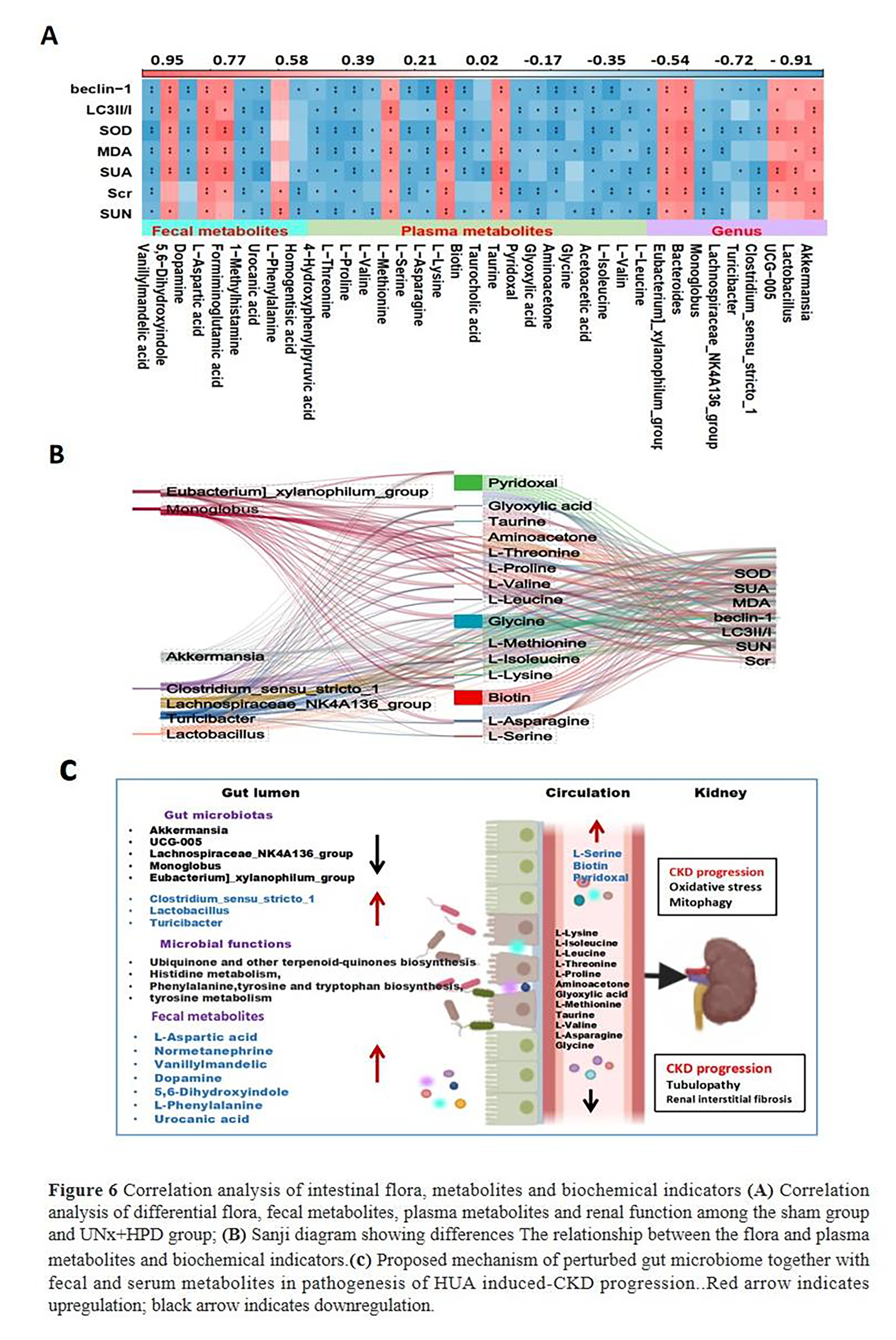

Supplement: Figure 6.tif [file IRNF_A_2387429_SM4133.tif]

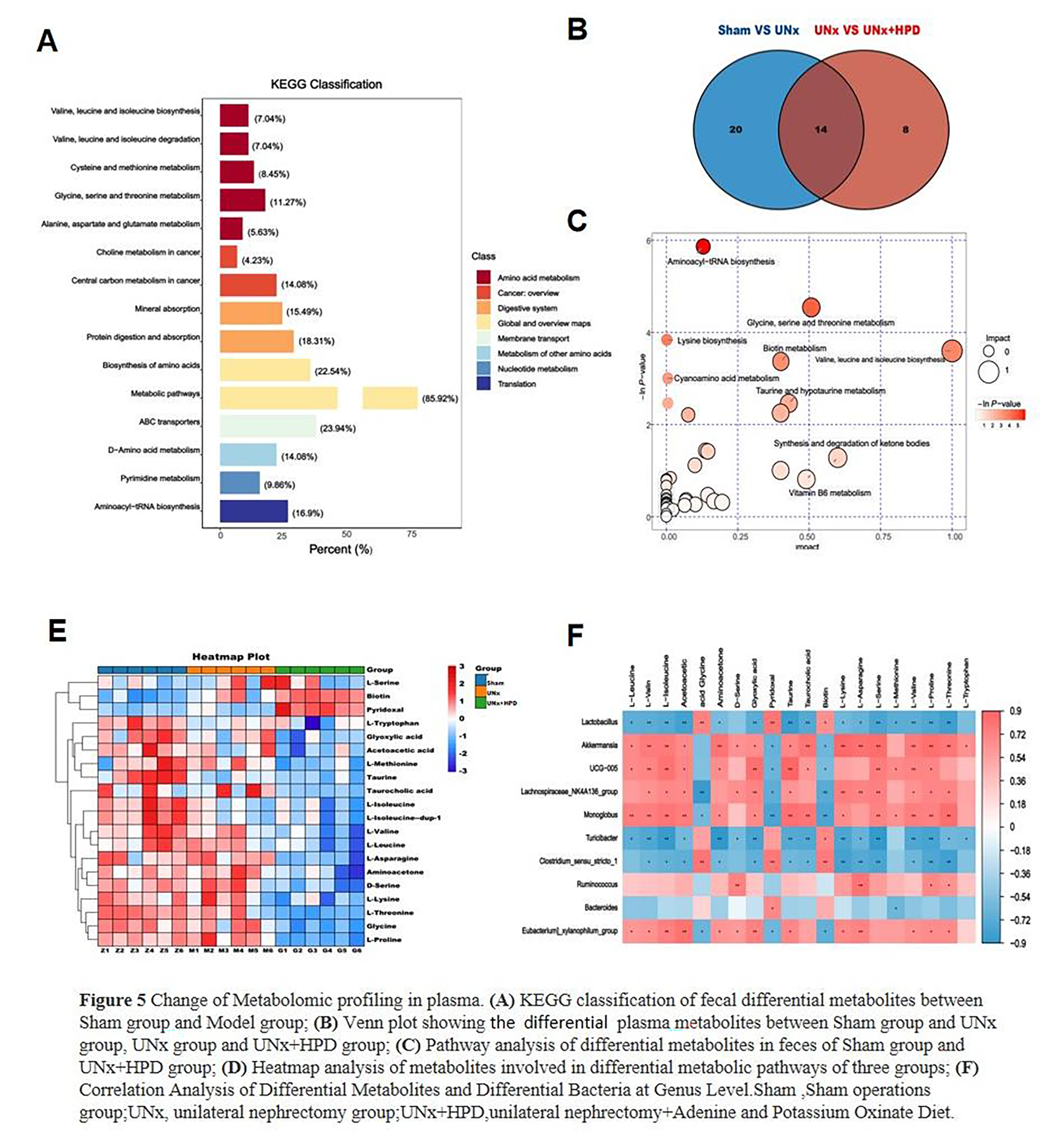

Supplement: Figure 5.tif [file IRNF_A_2387429_SM4132.tif]
